# Beyond Calcium and Vitamin D: Exploring Creatine, β-Hydroxy-β-methylbutyrate, Prebiotics and Probiotics in Osteosarcopenia

**DOI:** 10.3390/nu17142332

**Published:** 2025-07-16

**Authors:** José Eduardo Moreira-Velasco, Maria Fernanda Contreras-Alvarado, Hassan Rammal, Daniel Rivas, Gustavo Duque

**Affiliations:** 1 Geriatrics, Tecnologico de Monterrey, Monterrey 64700, Nuevo León, Mexico; ed.moreira94@gmail.com (J.E.M.-V.); drafernandacontreras@gmail.com (M.F.C.-A.); 2 Bone, Muscle & Geroscience Group, Research Institute of the McGill University Health Centre, Montreal, QC H4A 3J1, Canada; hassan.rammal@mail.mcgill.ca (H.R.); daniel.rivas@rimuhc.ca (D.R.); 3 Dr. Joseph Kaufmann Chair in Geriatric Medicine, Department of Medicine, McGill University, Montreal, QC H3A 2B4, Canada

**Keywords:** osteosarcopenia, sarcopenia, osteoporosis, nutraceuticals, supplements, geriatrics, microbiota, muscle, bone

## Abstract

**Background/Objectives**: Osteosarcopenia, the coexistence of osteoporosis and sarcopenia, in older adults, is an emerging geriatric syndrome linked to functional decline, increased frailty, and higher mortality. Evidence supports the benefits of interventions such as physical exercise and dietary supplementation with vitamin D, calcium, and protein in this population. Additionally, emerging supplements—such as creatine, β-hydroxy-β-methylbutyrate (HMB), probiotics, and prebiotics—are being investigated for their potential to enhance bone density, muscle mass, and physical function. This review aims to examine the current evidence on these novel nutritional strategies and provide a comprehensive synthesis of how these factors may synergistically influence both muscle and bone health. **Methods**: A comprehensive literature search was conducted across the PubMed/MEDLINE, Embase, Scopus, and Google Scholar databases. Relevant observational studies, clinical trials, systematic reviews, and meta-analyses published from January 2020 to June 2025 were included, and then a reverse search in the bibliography was used to expand on definitions and concepts. **Conclusions**: Nutritional interventions for osteosarcopenia play a pivotal role in not only improving bone and muscle composition but also enhancing functional outcomes in older adults. Emerging strategies involving creatine monohydrate, HMB, probiotics, and prebiotics show potential as part of a comprehensive patient-centered approach. However, further research is needed to determine the most effective strategies and to identify which patients are most likely to benefit from each supplement.

## 1. Introduction

Osteosarcopenia, defined as the concomitant presence of osteoporosis or osteopenia and sarcopenia, is an emerging geriatric syndrome associated with significant adverse health outcomes in older adults [1]. As the global population continues to age, with projections estimating 1.5 billion adults aged 65 and older by 2050 [2], the prevalence of osteosarcopenia is projected to rise, positioning it as a public health concern.

Osteoporosis is characterized by reduced bone mineral density (BMD) and microarchitectural deterioration, which significantly raises the risk of fragility fractures. The incidence of fractures is particularly high in older adults due to their concurrent increased risk of falls [3]. Sarcopenia, on the other hand, refers to the age-related loss of skeletal muscle mass, strength, and function. Currently, there is no approved pharmacological treatment for this condition [4]. When osteoporosis and sarcopenia coexist, they can synergistically exacerbate the risk of falls, fractures, functional decline, and mortality in older individuals [2].

The pathophysiology of osteosarcopenia is complex and multifactorial, involving shared risk factors and biological mechanisms. Age-related hormonal changes, chronic low-grade inflammation, oxidative stress, and protein metabolism disruptions all contribute to bone and muscle degeneration [1]. Current osteosarcopenia theories suggest that there is biochemical crosstalk through myokines and osteokines, as well as mechanical interactions between bone and muscle [5]. Therefore, a nutritional intervention that benefits one tissue may also influence the other through this crosstalk system.

Current interventions for osteosarcopenia primarily emphasize structured exercise programs and adequate calcium (1000–1200 mg/day), vitamin D (800–1000 IU/day), and protein (1.2–1.5 g/kg/day) intake [6]. However, emerging evidence suggests that novel nutritional strategies may offer additional benefits, including improvements in muscle mass, strength, fall prevention, and inflammatory modulation [7].

This review aims to explore the potential role of emerging nutritional supplements—including creatine, β-hydroxy-β-methylbutyrate (HMB), prebiotics, probiotics, and agents targeting bile acid metabolism—in supporting both bone and muscle health. By synthesizing the most recent scientific findings, this review seeks to inform comprehensive management strategies for osteosarcopenia and highlight areas for future research.

## 2. Materials and Methods

A comprehensive literature search was conducted across the PubMed/MEDLINE, Embase, Scopus, and Google Scholar databases. The MeSH terms: ‘osteosarcopenia’, ‘osteoporosis’, ‘sarcopenia’, ‘sarco-osteopenia’, ‘creatine’, ‘β-hydroxy-β-methylbutyrate’, ‘prebiotics’, ‘probiotics’, ‘bile acid metabolism’, and ‘nutritional supplements’ were used. OpenEvidence artificial intelligence was also employed in the literature search. Relevant observational studies, clinical trials, systematic reviews, and meta-analyses published between January 2020 and June 2025 were included. Additionally, a reverse bibliography search was conducted to further explore key definitions and concepts.

### 2.1. Study Selection

The selection of studies was based on their relevance to the topic, initially assessed through title and abstract, and subsequently confirmed through full-text review.

The inclusion criteria comprised published original research studies—specifically randomized controlled trials, cohort studies, case-control studies, and cross-sectional studies—that explored supplementation in osteosarcopenia. Literature reviews on sup-plementation in this condition were also included. We excluded studies that focused on medical (non-nutritional) interventions and case reports. Only English language publi-cations were considered.

### 2.2. Data Extraction and Synthesis

The reviewers conducted the database search and selected the studies to be included in this review. After identifying the relevant studies, the information was synthesized into themes and subthemes based on the supplements with the strongest supporting evidence.

### 2.3. Quality Assessment

Three reviewers evaluated each study’s methodology, sample size, and potential biases. The outcomes reported in each study were analyzed, and efforts were made to ensure that potential biases and limitations were adequately addressed in the review. Only studies with methodological quality and publication in high-impact journals were included.

## 3. Creatine

### 3.1. Creatine: A Well-Known Supplement

Creatine is a nitrogen-containing organic compound that plays a critical role in cellular energy metabolism. It serves as a short-term, localized energy reserve for adenosine triphosphate (ATP), particularly in tissues with high and fluctuating energy demands such as skeletal muscle and the brain [8]. Endogenously, creatine is synthesized primarily in the kidneys and liver via a two-step enzymatic process [9].

Dietary creatine is predominantly derived from animal-based foods, including meat and fish. In omnivorous individuals, approximately half of the body’s creatine requirements are met through dietary intake, while the remainder is synthesized from precursor amino acids. However, older adults often consume lower amounts of both creatine and its amino acid precursors, which may compromise creatine availability and suggest a potential benefit from supplementation to achieve optimal physiological levels [10].

Since the 1990s, creatine monohydrate supplementation has gained widespread popularity, particularly in sports and clinical settings. Extensive research has confirmed its safety profile. According to the International Society of Sports Nutrition, a daily intake of 3 g of creatine monohydrate is sufficient to maintain adequate creatine stores and is considered safe for long-term use in healthy individuals [11].

#### 3.1.1. Effects of Creatine in Aging Muscle

Supplementation with creatine increases intramuscular phosphocreatine availability, enhancing the capacity for ATP resynthesis during resistance exercise. This supports greater training intensity and volume, which in turn stimulates muscle protein synthesis and hypertrophy. Additionally, creatine may exert direct anabolic and anti-catabolic effects by modulating cellular hydration, influencing growth factor signaling (e.g., IGF-1), and reducing muscle protein breakdown [12].

Over the past few decades, research has consistently shown that creatine supplementation, combined with resistance training, yields greater improvements in muscle mass, strength, and functional performance in older adults compared to resistance training alone. Notably, functional gains have been observed through assessments such as the sit-to-stand test, indicating enhanced lower-body performance [7]. This is clinically relevant, as improvements in lower-limb muscle density are associated with a reduced risk of falls, functional impairment, disease progression, and premature mortality in the geriatric population [13,14]

#### 3.1.2. Effects of Creatine on Aging Bone

Bone cells rely on the creatine kinase reaction to generate energy, utilizing phosphocreatine to regenerate ATP [15]. This enzyme activity increases when osteoblasts become activated [16]. In vitro, studies have demonstrated that creatine supplementation enhances metabolic activity, promotes differentiation, and supports mineralization in osteoblast-like cells [17]. Activated osteoblasts also produce osteoprotegerin, a protein that acts as a decoy receptor for receptor activator of nuclear factor kB ligand (RANK-L). By blocking RANK-L’s interaction with RANK, osteoprotegerin inhibits the formation of bone-resorbing osteoclasts, thereby helping to reduce bone turnover [18].

While recent studies indicate that creatine may not significantly increase BMD in older adults [19], it could influence beneficial geometric changes in bone that enhance fracture protection [20]. Furthermore, the increased muscle mass often associated with creatine supplementation may indirectly stimulate greater bone formation over time by generating increased mechanical forces on the bone [21].

Collectively, creatine has the potential to reduce the risk of falls in older adults, which, in turn, decreases fracture risk [7]. Evidence suggests that these benefits are enhanced when creatine supplementation is combined with exercise interventions [21,22].

#### 3.1.3. Synergistic Effects of Creatine with Other Supplements

Combining creatine with other supplements may offer additional benefits. One study, for instance, found that healthy older men who took whey protein and creatine three times a week for ten weeks, alongside supervised whole-body resistance training, saw greater increases in whole-body fat-free mass and relative upper-body maximal strength than those who only took creatine [23].

Beyond protein, supplementing leucine and BCAAs (branched-chain amino acids) plus vitamin D has shown improvements in muscle mass, strength, and physical performance in older adults experiencing sarcopenia and malnutrition [24].

Interestingly, this review did not identify any studies that investigated the combined use of creatine with prebiotics or probiotics in older adults in relation to muscle mass, strength, or bone health. Given the potential synergistic effects, this represents a promising area for future randomized controlled trials. Similarly, while the role of creatine plus HMB has been explored in younger populations or athletes [25], its effectiveness when combined for older adults remains unestablished.

#### 3.1.4. Conclusions on Creatine

Given the positive findings regarding creatine’s effects on muscle and bone in healthy older adults, it holds potential as an adjunct to exercise training for managing osteosarcopenia. However, further investigation is needed to confirm its effectiveness in older populations.

## 4. β-Hydroxy-β-methylbutyrate

### 4.1. β-Hydroxy-β-methylbutyrate: An Emerging Supplement of Interest

HMB, a metabolite of the amino acid leucine, is gaining attention for its potential to impact muscle mass and function, especially in older adults. About 5% of leucine converts to HMB in the human body through enzyme-mediated processes [26]. Higher HMB plasma levels have been linked to increased appendicular lean mass and better hand grip strength (HGS) in healthy individuals [27].

HMB primarily affects muscle by boosting protein synthesis through the mTOR pathway stimulation, reducing protein breakdown, enhancing muscle repair, and improving aerobic capacity [28]. Long-term oral supplementation (1.5–3 g/day) appears safe for at least one year, with short-term dosing up to 6 g/day for 8 weeks showing no adverse effect [29].

#### 4.1.1. Effects of HMB on Aging Muscle

Older adults and individuals with frailty often experience a decline in physical activity, which accelerates muscle loss. Recent research indicates that HMB supplementation may mitigate lean mass losses in older inpatients, outpatients, and critically ill patients [26,30]. For patients with reduced mobility, HMB could be a valuable intervention to counteract these effects, improving quality of life and maintaining independence. However, other studies have yielded mixed results.

Several systematic reviews and meta-analyses have investigated HMB’s effects on muscle parameters. While certain trials report increases in lean soft-tissue mass (LSTM), others report no difference or insufficient evidence. A 2019 meta-analysis of 15 RCTs reported modest evidence supporting HMB’s role in increasing skeletal muscle mass and stronger evidence for improvements in muscle strength. However, this analysis included participants across all age groups, and the observed effect sizes were small [30].

A 2022 umbrella review analyzed 15 systematic reviews on HMB supplementation, using DXA-measured LSTM as a proxy for muscle mass. The findings were largely inconsistent: only five reviews showed some benefit for LSTM, while the remainder found no effect or lacked sufficient evidence. Of 12 studies assessing strength, only 4 showed some benefit, with most others indicating no effect or inconclusive results, particularly showing no consistent benefit in community-dwelling individuals [26].

More recent RCTs also present mixed results. A 2023 study involving older adults with sarcopenia reported significant improvements in HGS, gait speed, and muscle quality with HMB supplementation compared to placebo. However, no significant differences were observed in skeletal muscle mass or body composition parameters [28]. These findings are particularly relevant given that HGS is a core component in sarcopenia diagnosis [31].

A 2024 meta-analysis of six RCTs, specifically focusing on sarcopenia patients, found a statistically significant difference in HGS with HMB or HMB-rich nutritional supplements, but no significant differences in gait speed, fat mass, fat-free mass, or skeletal muscle index. This analysis suggested that HMB supplementation might be more effective with an intervention duration of 12 weeks or longer [32].

Finally, a 2025 meta-analysis of five RCTs revealed beneficial effects on muscle mass and strength, demonstrated by higher skeletal muscle mass index and elevated HGS in HMB intervention groups. The overall meta-analysis revealed an increase in HGS in the HMB intervention groups compared to the control groups. Nevertheless, no evidence of benefit on physical performance (as assessed by gait speed) was found [33].

#### 4.1.2. Effects of HMB on Aging Bone

Current evidence regarding HMB and bone health is limited to preclinical animal studies. For instance, in spiny mouse models of osteoporosis, HMB supplementation during pregnancy was linked to improved trabecular bone microarchitecture and the prevention of bone loss. This effect is likely due to HMB’s modulation of bone cell activity and collagen remodeling [34,35]. However, these findings have not yet been replicated in human studies, and no randomized controlled trials have evaluated HMB for osteoporosis prevention or treatment in humans.

#### 4.1.3. Synergistic Effects of HMB with Other Supplements

Recent research has begun to explore the potential synergistic effects of combining HMB with other nutrients:

HMB plus probiotics: Research suggests that certain probiotic strains might increase HMB exposure in tissues and plasma [36] or offer synergistic anti-inflammatory and antioxidant properties when combined with HMB [37]. Further research is required to fully investigate these mechanisms and benefits.

HMB plus Creatine: Several studies indicate positive effects on athletic performance when HMB is combined with creatine, although these studies have not specifically focused on older populations [25].

HMB plus Vitamin D: The co-administration of HMB-Ca with vitamin D3 in healthy older adults with documented vitamin D insufficiency has been shown to enhance muscle strength [38,39]

HMB plus Protein: HMB has been demonstrated to amplify the anabolic effects of plant-based (soy) protein ingestion during a fasting catabolic state [40]. Conversely, other studies in young athletes did not show additional improvements when HMB was added to whey protein [29].

#### 4.1.4. Conclusions on HMB

The evidence for HMB supplementation improving muscle mass, strength, and function in the geriatric population remains inconclusive. While certain studies report statistically significant outcomes, especially concerning muscle strength and quality, others find no significant effects or present inconclusive evidence. HMB supplementation appears to be safe and well-tolerated. Its potential as an option for managing age-related sarcopenia and osteoporosis is yet to be determined, and further research is crucial to establish HMB’s efficacy.

## 5. Prebiotics and Probiotics

### 5.1. Prebiotics and Probiotics: Targeting the Gut–Muscle–Bone Axis

Probiotics are described as “live microorganisms that, when administered in adequate amounts, confer a health benefit to the host” [41]. These typically include specific bacterial or yeast strains, most commonly from the *Lactobacillus*, *Lactococcus*, *Streptococcus*, and *Bifidobacterium* genera [42]. They are generally found in fermented foods or dietary supplements and are believed to exert their effects by competing with pathogens for adhesion sites, enhancing the gut barrier, modulating immune responses, and aiding in neurotransmitter production.

In contrast, prebiotics are non-digestible food components, often specific types of dietary fibers or oligosaccharides (such as inulin and oligofructose) that positively impact the host by selectively promoting the growth and/or activity of beneficial microorganisms [43,44]. These compounds act as substrates for these microbes, encouraging their growth and metabolic activity, which in turn can lead to host health benefits. They are considered safe for all ages after the fifth month of life, with side effects limited to bloating, gas, and increased bowel movements [45].

The term “synbiotic” refers to the combination of both, designed to enhance probiotic viability [46]. Currently, *Bifidobacterium* species are the most extensively studied in synbiotic applications related to bone health [47]. Modifying the gut microbiota through these interventions is under investigation.

The gut microbiota is acquired primarily from the mother at birth, then influenced by numerous factors, including genetic background, diet, age, medical treatments, antibiotic use, and geographical location [45,48,49]. Recent studies also indicate that myokines and cytokines, produced during skeletal muscle contraction and released into the bloodstream, can influence the gut microbiota. IL-6 was the first identified myokine, alongside others like irisin and myostatin. These molecules mediate metabolic processes, regulate inflammation, and influence muscle growth, contributing to overall health. Recent research has unveiled a bidirectional relationship between the gut and muscle, known as the gut–muscle axis. Exercise positively affects gut microbial diversity and composition through this axis, while the gut microbiota, in turn, impacts muscle function and metabolism. Evidence indicates that this muscle–gut axis plays a role in the pathophysiology of physical frailty and sarcopenia, although a definitive causal link has yet to be established [50]. Adverse effects are uncommon and usually mild when using standard commercially available products, often involving temporary gastrointestinal symptoms such as bloating, gas, or mild abdominal discomfort [51], although there have been reported cases of bacteremia and liver abscess associated with probiotic use in older patients, these are rare complications [52].

#### 5.1.1. Effects of Prebiotics and Probiotics on Aging Muscle

The gut microbiota, the most abundant and complex microbiota in the human body, is extensively studied for its association with various diseases [42]. It plays a pivotal role in the aging process by regulating energy balance, metabolism, and inflammation, thereby impacting the progression of sarcopenia [53]. Structural alterations in the gut microbiota are linked to the development of numerous chronic conditions, including inflammatory bowel disease, obesity, diabetes, and cardiovascular diseases [54]. The concept of the gut–muscle axis refers to a potential correlation between gut microbiota and the quality and functionality of skeletal muscle [55].

Currently, non-pharmacological interventions such as exercise and nutritional strategies remain the first-line treatments for sarcopenia. Developing innovative, safe, efficient, and affordable sarcopenia treatments remains a critical challenge [56].

According to the gut–muscle axis hypothesis, muscle function and metabolism significantly depend on the quantity and composition of the gut microbiota. Dysbiosis in the gut microbiota can lead to increased gut barrier permeability, endotoxin translocation, and insulin resistance, ultimately resulting in impaired muscle protein synthesis [56]. Consequently, gut microbes could emerge as potential biological targets for the prevention and therapy of muscle-related disorders like sarcopenia and muscle atrophy [53,57].

While *Lactobacillus* is commonly used for gut health and immune support, its precise mechanism in alleviating sarcopenia via the gut–muscle axis remains uncertain. However, mechanisms identified include the relief of inflammatory states, clearance of excess reactive oxygen species, improvement of skeletal muscle metabolism, and regulation of gut microbiota and its metabolites [56].

A comprehensive review in 2025 synthesized findings from ten studies (421 individuals with sarcopenia and 1642 control subjects) examining the association between gut microbiota and sarcopenia. The analysis revealed that individuals with sarcopenia exhibited an increased presence of certain inflammation-associated bacteria, such as *Proteobacteria* and *Escherichia*-*Shigella*. Conversely, there was a decreased presence of beneficial bacteria, including *Firmicutes*, *Faecalibacterium*, *Prevotella* 9, and *Blautia*. These alterations in gut microbiota may induce inflammation, impairing nutrient absorption, and disrupting metabolic processes [58]. Specifically in older adults, a significant decrease in the proportion of *Lactobacilli*, *Bacteroidetes*, and *Prevotella*, coupled with increased *Escherichia coli*, has been observed in sarcopenia patients [59]. *Lactobacillus* can restore the composition and beta diversity of intestinal microbiota, which may be one of the ways to play a therapeutic role in sarcopenia [60].

*Lactobacillus* alleviates oxidative stress by indirectly or directly inhibiting nitric oxide production. *L. plantarum* down-regulates the expression level of syncytin-1, nitric oxide synthase (iNOS), and the TNF-α gene present in skeletal muscle, restores overall energy balance in muscle tissue of animals, and reduces the oxidative response [61].

Sarcopenia is often accompanied by insulin resistance, and both are mutually pathogenic. *Lactobacillus* strains contribute to a decrease in insulin resistance [62]. Abnormal accumulation of lipids in the body will accelerate muscle atrophy and muscle steatosis [63]. Effects of probiotics in lipid metabolism include enhancement of lipid oxidation, reduction in white adipose tissue and fat accumulation, modulation of adiponectin (APN), and AMPK Pathways, increasing insulin sensitivity; these effects are highly strain-specific, and not all strains produce beneficial outcomes [56].

A comprehensive analysis of 22 studies investigating the effects of probiotics on sarcopenia across all age groups provides nuanced insights. Overall, the results showed no statistically significant improvements in muscle mass or lean body mass in individuals receiving probiotic supplementation compared to placebo [64]. Notably, only one study focused specifically on older adults aged over 70 years. In this trial, mildly frail participants supplemented with *L. plantarum* TWK10 for 18 weeks demonstrated significant improvements in HGS and muscle mass, though BMD remained unchanged [65]. These findings support the potential of probiotics to improve muscle function in sarcopenia, particularly in older adults, but emphasize the need for additional high-quality, targeted research to confirm these benefits and determine long-term effects.

#### 5.1.2. Effects of Prebiotics and Probiotics on Aging Bone

The gut microbiota significantly influences bone metabolism, presenting a potential new target to modify BMD. This interplay has led to the concept of the gut–bone axis [54]. Evidence primarily from mouse models demonstrates the gut microbiota’s involvement in modulating the interaction between the immune system and bone cells [45]. A recent meta-analysis further showed that prebiotics improve BMD in ovariectomized rats [66], highlighting an opportunity to address their role in osteosarcopenia.

Several pathways connect the gut microbiota to osteosarcopenia, including its interaction with calcium and vitamin D absorption. A reduction in 1,25-hydroxyvitamin D production can lead to gut inflammation and alter GM composition [67], hormonal secretion, and immune responses. For instance, oral supplementation with *L. reuteri* has been shown to increase serum 25-hydroxyvitamin D and tibial bone density in humans [68,69]. Similarly, *L. rhamnosus* strain GG and *L. plantarum* increased vitamin D receptor (VDR) protein expression [70]. Research has also found that *L. rhamnosus* GG and *L. plantarum* can increase the expression of the VDR protein in both mouse and human intestinal epithelial cells while enhancing its transcriptional activity. By activating the VDR signaling pathway, they ultimately promote vitamin D absorption and the expression of its target genes, such as the antimicrobial peptide cathelicidin, exerting anti-inflammatory effects [70].

Another key role is played by T helper 17 lymphocytes (Th17), a subset of proinflammatory T helper cells that play an important role in maintaining the mucosal barrier and preventing intestinal colonization by pathogenic bacteria [71]. Some bacteria, such as *Bacillus clausii* [72], *L. acidophilus* [73], and *L. rhamnosus* [74], enhance bone health by inhibiting osteoclastogenic Th17 cells and promoting anti-osteoclastogenic Treg cells in ovariectomized osteopenic mice. Th17 cells produce Interleukin 17 (IL-17), which exacerbates local inflammation, leading to increased inflammatory cytokines like TNF-α and IL-1, which, in turn, elevate RANK-L expression and activate osteoclast precursor cells and the RANK-L system [71]. Other important pathways include nucleotide-binding oligomerization domain proteins (NOD1, NOD2), which are intracellular sensors of pathogen-associated molecular patterns (PAMPs), mainly expressed on epithelial and immune cells, that bind bacterial peptidoglycans and activate the NFkB pathway, playing a key role in the effects of microbiota on bone. On the other hand, a recent study has identified Toll-like receptor 5 (TLR5) as a new mediator in the process of inflammation-induced bone loss and osteoclastogenesis, through the activation of the RANK-L pathway [71,75,76]

In preclinical research, using alginate oligosaccharides as a prebiotic significantly increased bone mass, boosted muscle function, and promoted gut barrier integrity in ovariectomized mice. This was achieved by modulating bile acid (BA) metabolism and reducing intestinal Th17 cells and peripheral inflammation [77]. Research using germ-free mice (raised in sterile conditions without gut microbiota) exhibits underdeveloped immune systems and shows varied bone density outcomes [45].

A potential mechanism by which the gut microbiota influences osteoporosis involves its effect on intestinal barrier function, particularly through the regulation of tight junctions, which are essential for maintaining barrier integrity. Disruptions in this barrier have been linked to glucocorticoid-induced osteoporosis [78]. In murine models, interventions such as *L. reuteri* and high-molecular-weight polymers (which enhance barrier function) have been shown to prevent glucocorticoid-induced bone loss. Immune cells in the intestinal subepithelial tissue produce osteoclastogenic cytokines like interleukin-1β and tumor necrosis factor-α (TNF-α), and increased intestinal permeability can elevate these cytokine levels, potentially leading to reduced BMD [79]. Additionally, the ratio of intestinal villus height to crypt depth is an important indicator of digestive and absorptive capacity. Studies have demonstrated that dietary supplementation with *Bacillus subtilis*, *Rhamnobacterium*, and heat-inactivated *L. plantarum* can increase this ratio, enhance mucosal morphology, support beneficial bacterial colonization, and ultimately improve nutrient absorption [80,81].

Gut microbiota can also affect bone remodeling through gut microbiota-derived metabolites, such as short-chain fatty acids (SCFAs) and trimethylamine N-oxide (TMAO). SCFAs, including acetic acid, propionic acid, isobutyric acid, butyric acid, isovaleric acid, and valeric acid, have been reported to play a significant role in osteoclasts and osteoblasts [82,83]. TMAO, a choline metabolite, is another important gut microbiota-dependent metabolite associated with bone remodeling. Serum TMAO levels in osteoporosis patients are often higher than in healthy individuals, showing a negative correlation with BMD [84]. SCFAs may also influence bone health by affecting the production of hormones such as parathyroid hormone, insulin-like growth factor-1 (IGF-1), and serotonin [54].

Regarding endocrine regulation, the intestine is considered the largest endocrine organ in the human body [85]. Cortisol can regulate immune cell activity and cytokine release in the intestine, thereby affecting intestinal permeability, gut barrier function, and GM composition.

#### 5.1.3. Gut Microbiota and Bile Acid Metabolism

Bile acids (BAs) are a group of cholesterol metabolites, an important component of the bile. Once considered solely digestive agents, they are now recognized as multifunctional signaling molecules with effects on metabolism, the immune system, and intestinal bacterial growth [86,87].

They are classified into primary and secondary BAs based on their site of synthesis and degree of microbial modification. Primary BAs are synthesized in hepatocytes; the two main primary BAs are cholic acid (CA) and chenodeoxycholic acid (CDCA). Primary bile acid supplements are used to treat cholestatic liver diseases to replace or augment endogenous bile acids. These acids are conjugated with glycine or taurine and are then secreted into the bile and stored in the gallbladder. After food intake, they are released into the duodenum. About 95% of primary BAs are reabsorbed, but the remaining 5% reach the colon and are modified by the gut microbiota into secondary BAs, mainly deoxycholic acid (DCA) and lithocholic acid (LCA). These modifications alter the individual’s BA pool. Variations in BA composition have been associated with human longevity [87]. This relationship may play a role in mitigating the development of age-related diseases, particularly in connection with microbiota dysbiosis.

BAs are believed to be involved in the pathogenesis of secondary sarcopenia, promoting skeletal muscle growth while reducing skeletal muscle waste. Systemic circulation allows the secondary BAs to act as endocrine molecules [88]. *Lactobacillus* can modulate BAs metabolism and affect its gastrointestinal transport [89]. Also, metabolomic studies have identified disturbances in and BAs metabolism in patients with osteoporosis [90,91].

A cross-sectional study in China evaluated the relationship between serum BAs levels and BMD in 150 postmenopausal women, stratified into three groups: osteoporosis, osteopenia, and healthy controls. Researchers measured serum concentrations of total BAs, fibroblast growth factor 19 (FGF19), bone turnover markers, and BMD. Findings revealed that women in the osteoporosis and osteopenia groups had significantly lower serum BAs levels compared to healthy controls, and BAs concentrations were positively correlated with BMD at the lumbar spine, femoral neck, and total hip regions [92].

BAs are increasingly recognized as modulators of bone and muscle, since their metabolism appears to affect them both directly as endocrine molecules and indirectly through interactions with gut microbiota. This presents an interesting new pathway to explore in the treatment of osteosarcopenia.

#### 5.1.4. Conclusions on Prebiotics and Probiotics

**Figure 1:**
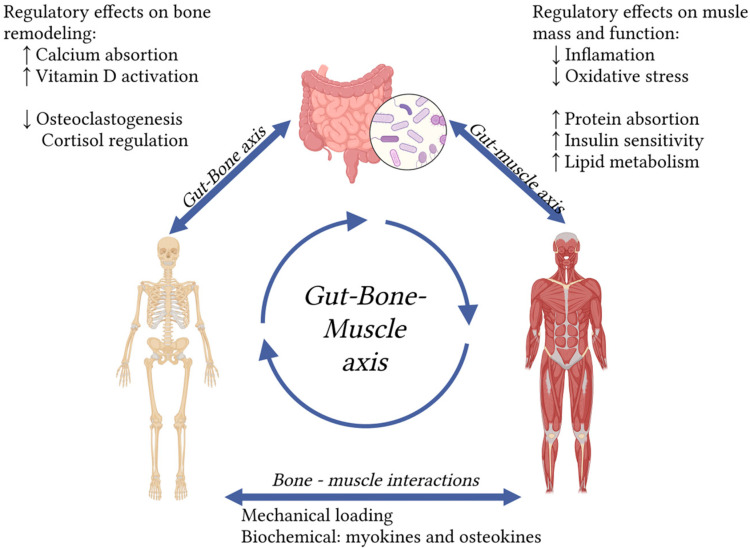
The gut–bone–muscle axis highlights the impact of gut microbiota on osteosarcopenia. Arrows indicate interactions. ↑ indicates an increase; ↓ indicates a decrease. (created in https://BioRender.com).

Literature describes the “gut–bone axis” and the “gut–muscle axis” as distinct but related concepts, both focusing on the profound influence of gut microbiota on bone and muscle health. Taken together, these results highlight that probiotics and prebiotics can positively regulate both muscle and bone. Considering the strong association and shared pathophysiology between osteoporosis and sarcopenia, the overarching concept of a gut–muscle–bone axis (Figure 1) presents significant potential for future research and therapeutic strategies targeting osteosarcopenia.

## 6. Discussion

**Figure 2:**
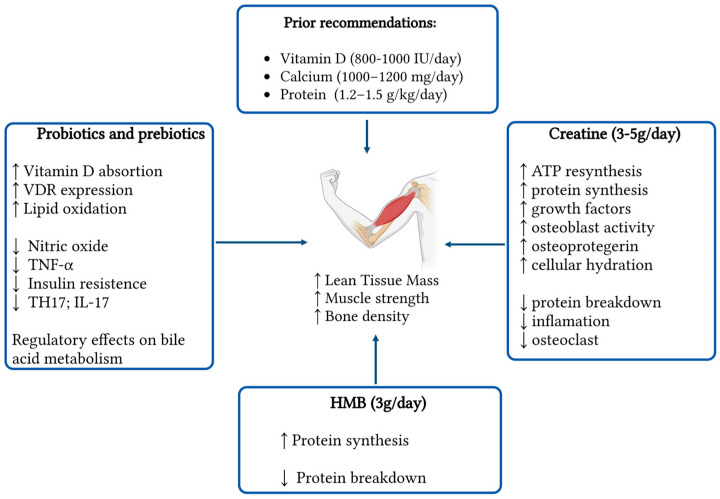
Summary of nutritional supplements in osteosarcopenia. Arrows indicate interactions. ↑ indicates an increase; ↓ indicates a decrease. (created in https://BioRender.com).

Searching for new strategies to treat and prevent osteosarcopenia is needed due to the upcoming shift in the geriatric population. In this review, we conducted a comprehensive analysis of some of the emerging supplements that can be used and how these affect the muscle and bone (Figure 2). Overall, they appear safe and well tolerated in the older population with minimal risks of adverse effects. Creatine monohydrate has been studied in multiple scenarios across time, with the main action over the muscle and indirectly promoting bone health. These positive changes appear tightly linked to routine exercise, highlighting the importance of a comprehensive and multidisciplinary therapy. The literature supports the use of creatine plus protein and vitamin D [6], giving it an advantage over other supplements yet to be studied in combination.

The body of evidence supporting HMB supplementation continues to grow, although results remain heterogeneous. Most studies have focused on its effects on muscle health, given its role in stimulating protein synthesis, rather than on bone health. Interestingly, while some trials report less favorable outcomes in terms of body mass composition, improvements have been observed in strength measures such as HGS. Since long-term HMB supplementation is safe, a more integrative evaluation considering its impact on functional outcomes may offer a clearer understanding of its real-world benefits.

Probiotics and prebiotics represent promising interventions for maintaining a healthy gut microbiota, with potential benefits not only for osteosarcopenia but also for other chronic conditions associated with aging. However, several considerations must be addressed regarding their supplementation. First, an individual’s microbiota composition is heavily influenced by environmental and personal history factors, which makes it challenging to generalize findings across diverse populations. Additionally, different bacterial strains appear to confer benefits to specific physiological systems, raising important questions about strain selection and how to translate this knowledge into clinical practice. Bile acid metabolism, which closely interacts with gut microbiota, has emerged as an area of research in the geriatric population. While clinical evidence for probiotics is more robust, further exploration of prebiotics—and the combined use of both—offers valuable opportunities for future investigation.

## 7. Conclusions

Nutritional interventions for osteosarcopenia play a pivotal role not only in improving bone and muscle composition but also in enhancing functional outcomes in older adults. Emerging strategies involving creatine monohydrate, HMB, probiotics, and prebiotics show potential as part of a comprehensive patient-centered approach. However, further research is needed to determine the most effective strategies and to identify which patients are most likely to benefit from each supplement.

## Data Availability

No new data were created.
